# The social construction of teacher and learner identities in medicine and surgery

**DOI:** 10.1111/medu.14727

**Published:** 2022-01-26

**Authors:** Peter Cantillon, Willem De Grave, Tim Dornan

**Affiliations:** ^1^ Discipline of General Practice National University of Ireland Galway Ireland; ^2^ School of Health Professions Education Maastricht University Maastricht The Netherlands; ^3^ School of Medicine, Dentistry and Biomedical Sciences Queens University Belfast Belfast UK

## Abstract

**Introduction:**

There are growing concerns about the quality and consistency of postgraduate clinical education. In response, faculty development for clinical teachers has improved formal aspects such as the assessment of performance, but informal work‐based teaching and learning have proved intractable. This problem has exposed a lack of research into how clinical teaching and learning are shaped by their cultural contexts. This paper explores the relationship between teacher–learner identity, educational practice and the workplace educational cultures of two major specialties: internal medicine and surgery.

**Methods:**

This was a secondary analysis of a large dataset, comprising field notes, participant interviews, images and video‐recordings gathered in an ethnographic study. The lead author embedded himself in four clinical teams (two surgical and two medical) in two different hospitals. The authors undertook a critical reanalysis of the observational dataset, using Dialogism and Figured Worlds theory to identify how teachers and postgraduate learners figured and authored their professional identities in the specialty‐specific cultural worlds of surgery and internal medicine.

**Results:**

Surgery and internal medicine privileged different ways of being, knowing and talking in formal and informal settings, where trainees authored themselves as capable practitioners. The discourse of surgical education constructed proximal coaching relationships in which trainees placed themselves at reputational risk in a closely observed, embodied practice. Internal medicine constructed more distal educational relationships, in which trainees negotiated abstract representations of patients' presentations, which aligned to a greater or lesser degree with supervisors' representations.

**Conclusions:**

Our research suggests that clinical education and the identity positions available to teachers and learners were strongly influenced by the cultural worlds of individual specialties. Attempts to change work‐based learning should be founded on situated knowledge of specialty‐specific clinical workplace cultures and should be done in collaboration with the people who work there, the clinicians.

## Abstract 2

Cantillon et al. explore how clinical teaching and learning are uniquely shaped by the specialty‐specific cultures of postgraduate training.

## 1 INTRODUCTION

The widespread adoption of competency‐based curriculums in postgraduate medical education (PGME) has greatly increased the expectations and responsibilities of clinical teachers.^1^ In response, there has been an ‘exponential’ increase in faculty development (FD).^2^ Despite FD efforts to professionalise clinical education, there remain worrying inconsistencies in the quality of clinical supervision and the adjudication of learners. Moreover, there are growing concerns that trainees are not being given sufficient opportunities to participate meaningfully in practice.^2^, ^3^, ^4^ It is clear that the educational effects of credentialing teachers and providing standalone teaching skills workshops have neither transferred well into practice, nor proved sustainable.^5^, ^6^, ^7^, ^8^, ^9^ This disappointing impact has been attributed to cultural and organisational factors features of clinical workplaces that undermine teacher development.^6^, ^7^ These problems are compounded by a lack of empirical research into how social and cultural contexts shape the practices of teaching and learning.^10^, ^11^

The relationship between teaching, learning and cultural context aligns better with sociocultural theory than the cognitive models that underpin much FD scholarship.^12^ From a sociocultural perspective, clinical education is a process of participating in the shared activities of institutions such as hospitals and clinical teams, and ‘becoming’^13^ a person whose identity is forged in particular cultural contexts.^14^, ^15^, ^16^, ^17^, ^18^ There has been little research into how identity formation, clinical supervision and the influence of cultural context relate to one another.^19^ Self‐report studies have shown how postgraduate trainees master shared practices, rules of thumb and embodied understandings,^20^, ^21^, ^22^, ^23^, ^24^ but such methodologies have been largely insensitive to the implicit effects of cultural context.^25^, ^26^ Observational studies have shown how graduate learners seek legitimacy in clinical teams by reproducing socially sanctioned token behaviours, but not how social and cultural contexts shape the roles of teachers and learners.^27^, ^28^, ^29^, ^30^, ^31^, ^32^, ^33^, ^34^, ^35^

In response, we launched a programme of research to develop a situated understanding of the relationship between clinical teaching, learning and social context. Our purpose was to develop a theoretical framework that would inform future contextually sensitive FD initiatives. An ethnography of four hospital teams (two internal medicine and two surgical) in two separate teaching hospitals over a period of 1 year (2016–2017) yielded a rich dataset.^19^ We used Goffman's dramaturgical theory, to explore the relationship between teacher and learner identity, educational practice and ways of talking and acting that typify participation in clinical teams. This showed that clinical teachers embodied rather than articulated their teams' implicit curriculum of norms and expectations. Trainees responded by reproducing teachers' embodied standards to create impressions of themselves as capable team participants.^19^ This methodology proved insensitive, however, to the shaping effect of specialty‐specific culture on teaching and learning. The aim of this paper was to reanalyse the observational data using a theoretical framework, Figured Worlds, to identify how the contrasting cultures of two major specialties, surgery and internal medicine (IM), influenced teachers' and learners' identity formation. The research question was as follows: How do the identities of teachers and learners interplay with one another in the cultural worlds of specialty clinical teams where the linked practices of working, learning and teaching are situated?

### 1.1 Theoretical framework

Figured worlds is a critical theory that provides conceptual and analytical tools to examine relationships between identity formation and culture.^36^ A figured (or cultural) world is a socio‐historical context (e.g. membership of a clinical team) that affords particular experiences, ways of talking and acting and social possibilities.^16^ Identity, within Figured Worlds, is not possessed; it is dynamic and evolving, constructed by speech and other symbolic acts, finding form in the interactions between individuals and the cultural worlds that they live and work in.^37^ Drawing on Bakhtin's theory of Dialogism, Figured Worlds assumes that speech and other symbolic acts give individuals agency to self‐author identities in cultural worlds.^15^, ^16^, ^38^ In Figured Worlds, the term ‘figure’ refers to the identity possibilities that are embodied by, for example, teachers from whom residents learn.^36^ The term ‘figuring’ superficially resembles role modelling but differs in how it places greater emphasis on learners' agency in forging their own identities, rather than assimilating roles.^36^, ^37^, ^38^ In this account, when we use the term ‘modelling’, we are referring specifically to the actions of teachers in demonstrating skills or articulating thinking for learners.^39^ We also differentiate between modelling and coaching where ‘coaching’ describes when teachers' observe learners' performances and provide feedback to support growth and development.^40^

Figured worlds acknowledges that high‐level institutional and sociohistorical discourses (which Gee terms big‐D Discourses) structure cultural worlds, create social positions, and make those positions more or less available to people with different identities.^41^ Importantly, Figured Worlds also allows scholars to reveal how the contents of everyday speech (little‐d discourse) give people agency to resist the structuring effect of big‐D Discourses.^36^, ^41^ In this way, the everyday use of (small‐d) discourse constructs and reconstructs (big‐D) Discourses.^41^ In this study, we used Figured Worlds theory's linguistic concepts to make sense of our ethnographic observations of cultural worlds in action (e.g. the educational rituals particular to the cultures of surgery and IM) and everyday speech (discourse), which give individuals agency to navigate cultural worlds.

## 2 METHODS

### 2.1 Setting and sample

This article reports a secondary analysis of an ethnography, carried out to explore how clinical education is actualised in the day‐to‐day working and learning environments of a surgical and an IM clinical team, in each of two separate teaching hospitals in Ireland. The hospitals were of similar size covering largely urban populations in two adjacent Irish cities. We selected surgery and IM because they represent practices within the same profession whose socio‐historical origins are very different. Although the sampling strategy was chosen for dramaturgical research, it was well suited also to exploring the influence of cultural factors on identity formation in medical education.

A ‘gatekeeper’ in each hospital distributed a leaflet giving information about the study amongst all clinical teams. The lead author (PC) then spoke about the study at medical and surgical grand rounds where most teams were represented. He invited offers to participate and included teams in the order they volunteered. Those included were typical of clinical team units in Ireland in that they were composed of one or more lead consultant specialists, one or two senior specialist registrars (senior residents) and two to four junior trainees (junior residents).

### 2.2 Research ethics approval and protection of participant identity

The ethnographic design and its associated data collection methods were approved by the research ethics committees of the two participating teaching hospitals (references: C.A. 1150 and 062/16). All participating team members received an information sheet about the research including the intention to publish the results. All provided written consent to participate in the research. Any patients or health care staff who were incidentally included in video or image data received an information sheet with researchers' contact details, which they were asked to read before giving verbal consent to be included in the dataset. If such consent was not forthcoming, we deleted the relevant data segments.

### 2.3 Data collection

The lead author (PC) embedded himself as a marginal participant observer^42^ (i.e. he observed, and at times partook in activity) in each team for between 12 and 16 weeks in 2016–2017. He attended each team's activities for approximately 2 days each week including ward rounds, multidisciplinary team meetings, surgical theatre, coffee breaks and formal educational events such as grand rounds. He collected data in multiple formats including contemporaneous field notes, digital images, video‐recordings of out‐patient encounters and exit interviews with all participants. At the end of each day of observation, PC developed his contemporaneous field notes into rich descriptions of what he had observed. Exit (semi‐structured interviews) and teacher–learner interactions captured on video were transcribed verbatim and pseudonymised. Likenesses of individuals captured in digital images and video stills were disguised and rendered unidentifiable using Akvis Sketch 23.5 software.^43^

### 2.4 Data analysis

Our orientation toward Figured Worlds empowered us to assume that analysing language would show how cultural worlds privileged particular ways of talking and behaving and would influence how individuals could form identities within those worlds. Two important Figured Worlds concepts provided analytical tools with explanatory power.^36^ ‘Positioning’ refers to how other people's speech grants or withholds identity possibilities.^16^, ^18^ ‘Self‐authoring’ refers to how individuals incorporate other people's speech into their own speech acts to negotiate, create or contest positions.^36^

The dataset included field notes from 640 hours of observation, transcripts of 34 exit interviews with participating team members (16 surgical and 18 IM), 30 hours of out‐patient video‐recordings and digital images provided by in‐the‐field photography. NVivo 12 software (QSR international 2018) facilitated analysis, which moved iteratively between original data and representations of the data, using memoing techniques to capture emerging interpretations. PC led the analysis and wrote the memos. TD and WdG met regularly with PC to review the interpretation.

The first phase of analysis, which treated the surgical and IM datasets as two distinct figured worlds, systematically sought key components of Figured Worlds,^36^, ^41^ which we list in Table 1. In practice, this meant reading and rereading field notes, interviews and video transcripts to identify meaningful acts, figured types (e.g. teachers in a particular specialty), culturally significant artefacts and other genres listed in Table 1. We regarded interpretive inferences as valid when linguistic evidence, symbols or behaviours present in video or digital images provided empirical support.^41^ We used this evidence to examine how participants' communicative actions ‘orchestrated’^36^ their identity positions.

**TABLE 1:** Key components of figured worlds used in the analysis

FW components	Meaning	Examples in surgical and IM team activities
Meaningful acts	Self‐evident behaviours, rituals and events.	Prototypical events: e.g. case presentations, ward rounds, case conferences.
Figures	Persons fulfilling prototypical roles	Teacher as coach; teacher as model; self‐directed learner.
Artefacts	Regularly encountered resources with cultural meaning.	The patient's body in surgical contexts Cognitive representations of the patient in IM
Figured language	Typical linguistic and narrative strategies.	Regular ways of talking and narrating in surgery and IM.

The second phase of analysis identified patterns of ‘identity work’ (i.e. the negotiation of identity positions in social contexts) that characterised the different worlds of surgery and IM. We used field note observations, excerpts of spoken language and video transcripts to synthesise a narrative summary of the cultural worlds of surgery and IM.

### 2.5 Rigour and reflexivity

Sensitised by Figured Worlds, we used rich description, crystallisation and multi‐vocality^44^, ^45^ to move from raw data to interpretation. We agreed interpretations between two people who had independently read the data.^44^ We strengthened the trustworthiness of our interpretation by seeking evidence from more than one source: for example, field note and interview data repeated over time.^45^ We applied the Figured Worlds concept of multi‐vocality by seeking similarities and differences between different individual participants and different teams in the same discipline.^36^ We sought participants' opinions about the validity of our interpretation in two ways: first by agreeing interpretations with specialty‐specific groups of participants and second by showing individual participants samples of original data and our interpretations of them. These validity negotiations refined our interpretations and conclusions.

In keeping with best ethnographic research practice,^42^ we used our insider (PC: general practitioner; TD: hospital specialist) and outsider (WdG: educational psychologist) perspectives to scrutinise otherwise taken‐for‐granted aspects of language and practice. PC kept a reflexive diary to ensure that the relationship between personal perspectives, observations and interpretations was available for scrutiny.

## 3 RESULTS

Becoming a surgeon or a physician was shaped by culturally valued ways of knowing and acting peculiar to the cultural worlds of surgery and IM and was inextricably linked to their specialty‐specific practices.

### 3.1 Core artefacts and practices in surgery

The core practice of surgery was identifying and correcting anatomical manifestations of disease. Proximal, coaching relationships between teachers and their protégé learners centred on the dominant cultural artefact: the patient's body. The surgical learning culture was more confrontational than the culture of IM. Surgeons observed trainees closely and scrutinised their practice, particularly in theatre.

Oh it's shocking the level of scrutiny because when you scrub in you are conscious of your surroundings. When you start off, it's the way you hold stuff, the way your surgeon is looking at you your senior colleagues are looking at you, anaesthetists are looking at you, and forming opinions about you and the nurses are too. (H1 T2 resident interview)

### 3.2 Core artefacts and practices in IM

In IM, the most important cultural artefacts were patients' clinical records and cognitive representations of their presentations as communicated and negotiated between team members. Here, the core practice was creating and manipulating plausible abstract representations of how patients had presented. These representations informed teacher–learner relationships, which were more distal than relationships in surgery. Given the largely opaque, cognitive nature of IM practice, teachers framed teaching as verbalising their thinking and acting in ways that trainees could recognise and reproduce. When talking about his habit of repeatedly checking during case presentations whether trainees had explored their patients' social and past histories, for example, one consultant explained: ‘What I'm hoping to transmit is that these are the important questions you need to ask’ (H1 T1 IM consultant 1 interview). IM physicians' talk emphasised the need for learners to be self‐directed and independent. For example, a consultant conceptualised postgraduate clinical education on the wards as follows: ‘they [the trainees] need to be taking responsibility. You have to let them learn by themselves … They need to learn and show learning themselves’ (H1 T1 IM consultant 2 interview). The emphasis in IM on modelling thinking and encouraging trainees to learn self‐directedly contrasted strongly with the intense observation and coaching of trainees' practice, which characterised surgical education.

### 3.3 Comparing the cultural worlds of surgical and IM teams

Table 2 illustrates ways of knowing, seeing, talking and being that were particularly valued in the cultural worlds of surgery and IM. Lead clinicians figured and articulated the dispositions and capabilities shown in Table 2, which trainees used to position themselves as capable participants in their respective specialties.

**TABLE 2:** The cultural backdrop for figuring and self‐authoring in internal medicine and surgical teams

Valued ways of:	Cultural world of surgical teams	Cultural world of internal medicine teams
Knowing	Surgeons valued categorical knowing, i.e. knowledge founded on fundamental anatomical and pathophysiological principles. For example a consultant figures surgical knowing for medical students: ‘if the patient has infection in solid tissue it's an abscess, a similar infection in the bloodstream is septicaemia’ (H2 T2 Field note). Categorical knowing helped surgeons to limit their scope of practice and constituted what surgeons termed a ‘foundational understanding’ that underpinned each surgeon's basis for knowing and acting. Foundational understandings informed ‘surgical intuition’, a rapidly available form of knowing that enabled surgeons to act in situations of incomplete information or urgency. Whereas exchanges between surgeons were often robust, they were careful not to undermine each other's foundational understandings. For example, a surgical supervisor figures etiquette in mutual positioning amongst surgical colleagues: ‘The surgeon develops an understanding. The surgeon applies that understanding and their results are linked to that particular understanding. That understanding becomes their foundation. If you were to disrupt that understanding or that foundation, then you are disrupting a very core process in them (H2T2 Consultant 2 interview).	Internal medicine knowing was valued in terms of its quantity, (knowing lots), its quality (logical and evidence‐based) and its applicability (flexible implementation). For example, a medical consultant uses a role model narrative to figure internal medicine knowing: ‘He seemed to know everything. He was just a genius. He knew everything about every speciality. He would hold the grand rounds every week and bring in these really exotic, complicated cases that had great clinical signs and he was a real master of general medicine’ (H2T1 Consultant interview). Knowing like an internal medicine doctor enabled physicians to create coherent abstract representations of complex patient presentations that informed subsequent diagnostic, therapeutic and prognostic decisions. Abstract representations of patient presentations were judged in terms of their internal consistency, their logic and their alignment with the existing evidence base. ‘I would say two or three interactions with a junior doctor will tell me their knowledge, their ability to assimilate information, the right information …. and it is quite obvious the ones who do not. There is no overall picture forming, it's like they are check listing questions, but they do not know what to do with the information, they are not collating it’ (H1T1 Consultant 2 interview).
Seeing	A surgical way of seeing privileged surgical practice by focusing on the functional outcomes of surgery rather than the scarring or disfigurement associated with surgical intervention. ‘This woman has a lovely stoma for us to look at’ (H2T2 Field note Resident talking to medical students). A surgical way of seeing marginalised delicacy or embarrassment about bodies, odours, discharges etc. ‘Nobody squeamish? We are all clinicians here and we are not afraid of bodies!’ (H2 T2 Fieldnote Consultant talking to surgical team).	An internal medicine way of seeing privileged clear‐sightedness and gestalt in the context of complex patient presentations. ‘This man was sent in by his GP with what he thought was a lower respiratory tract infection. However, when I talked to him it became clear that his problem was not cough, but shortness of breath, particularly at night. He needed a lot more pillows and found it very hard to lie flat … Now, you will never really get a clearer history than that of paroxysmal nocturnal dyspnoea’ (H2T1 Field Note Consultant talking to his team at the patient's bedside). An internal medicine way of seeing privileged the ability to identify salience in a mass of patient historical and investigative detail.
Talking	Surgical talk was unadorned, pragmatic language that conveyed identification with a surgical perspective. For example, a surgical resident figures a surgical way of narrating a case: ‘She had a big ovarian cyst. When we were trying to remove it, it burst scattering crap all over the abdomen’ (H1T2 Field notes Resident talking). Surgeons used ‘hero’ disaster‐deliverance narratives to position themselves as capable: ‘She came to us with an abdominal fistula. She was shedding raw faeces all over her abdomen wall and it was getting very excoriated. We decided to have a go at fixing this. We went in and eight hours later we closed up. A few days later a new fistula opened above the old one. However, this was a lot less painful and problematic than the old one’ (Fieldnote H2 T2 Resident talking).	Physicians talk foregrounded precision, logic and coherence to present compelling and satisfying abstract models of patient cases for a physician audience. ‘You know if someone is capable … There are different ways in which people present cases for example. So you come in to do a post call ward round and somebody says “this patient came in, and they presented with shortness of breath, and they had a cough, and this is the x‐ray.” Or “this patient came in with exacerbation of COPD, and we have done the following and this is the chest x‐ray.” You know from their way of managing things’ (H1T2 Consultant 3 interview).
Prudence	Being prudent like a surgeon meant navigating the tension between caution and action. For example, a surgical resident self‐authors as a prudent surgeon when discussing therapeutic options with a patient. ‘I think you have a hernia there. These are a very common thing that happen after major surgery like you have had. They are weaknesses in the belly wall and sometimes the gut pushes through like this. Yours has a wide neck and is quite small. I do not think it's going to get into any trouble …. Surgery would mean opening up your belly again and putting in gauze like stuff to hold it together – I do not think you want any more surgery do you?’ (H2 T2 Video transcript or Resident OPD consultation).	Being prudent like an internal medicine physician meant being reflexive and circumspect in relation to extant ideas, as well as new information or data. For example, an internal medicine physician figured internal medicine prudence as follows: ‘There are many ways to skin a cat, and medicine will make a liar of you because you do not know the right answers. It's not a science. So much of it is how the story is told [and] whether you have the ability to go back and readdress what you did on the first day. Was it the right thing to do and are you prepared to change your plan?’ (H1T1 Consultant physician interview).
Resilience versus self‐directedness.	Being resilient was highly valued in surgical team culture. Surgical resilience meant being capable of normalising postoperative complications, justifying actions and attributing poor outcomes to factors other than self. ‘Complications happen; they just happen, and I feel that you cannot get too bothered by it, because if you get too bothered by it, the next patient is affected. You process it, leave it in that room and you move on …. I have had to go away pretend nothing has happened’ (H2T2 Resident interview). Being resilient as a surgical trainee meant deflecting reputational threat by choosing to interpret critical comments from supervisors as coaching interventions rather than attacks on personal capabilities.	Being self‐directed was highly valued in internal medicine team culture. Being a self‐directed learner meant observing and absorbing supervisors' practice and being motivated to learn for oneself. An internal medicine emphasis on self‐directedness favoured a modelling approach to clinical education as opposed to the more coaching orientated approach prevalent in surgery. Here an IM consultant figures the modelling teaching approach of IM: ‘You lead by example, and you hope that people will watch what you do and if you do it well they will derive a positive experience from it. I do not think doctors need to be spoon fed. You're relying upon self‐directed learning’ (H2T1 Consultant interview).

### 3.4 Identity work in formal educational events

#### 3.4.1 Surgical grand rounds

Surgical grand rounds were educational events in which, each week a pre‐selected surgical team narrated interesting or challenging cases from the previous month to a critical audience of fellow surgeons and trainees. The surgical grand rounds ‘game’ required a resident to figure his or her surgical team as capable by confidently presenting cases in an intense conference environment. Members of the presenting team were subjected to pointed and at times aggressive questions from members of the audience during surgical case presentations. Questions were expressed in ways that positioned questioners as capable, while putting the presenting team on the defensive: ‘The staging criteria you use for prostatic cancer are very outdated compared to those we use in breast cancer and why are you still doing those “trans‐faecal” biopsies? They have a serious infection rate?’ (H1 T2 Field notes).

Surgeons subjected audience members to rapid and challenging question strategies that often carried considerable risk of reputational damage: ‘Okay what's going on here? Come on everyone it's obvious. Is anyone going to have a go?’ (H2 T2 Field notes). Surgeons authored the combative and challenging behaviours characteristic of surgical grand rounds as an appropriate way of preparing future surgeons to think on their feet.

You could make an argument that a nice relaxed environment is conducive to good education, but we are responsible for preparing them for professional activity and professional activity is a battle ground. Every moment of every day they are going to be faced with challenges. So, what I try to do is to get them thinking surgically whilst being challenged. H2 T2 Consultant interview

#### 3.4.2 Internal medicine case conference

Medical case conferences were a weekly educational event in which the ‘on’ IM team, nominated a resident to present ‘interesting cases’ they had managed in the previous few months. The climate of these conferences was more formulaic and discursive than in surgical grand rounds. For example, junior residents always presented cases using a standardised structure. ‘She presented a 57‐year‐old patient with aspergillosis. She outlined the presenting complaint, the past history, the medical and surgical histories, the systems review and the medication list’ (H2T1 Fieldnotes). Unlike surgical grand rounds, the presentation was not interrupted by anyone in the audience until the senior physician convenor invited questions or comments. Whereas the conference environment was considerably less pressurised than surgical grand rounds, questions from supervisors could still be perceived as posing a threat to residents' reputations. Residents frequently responded to questions by positioning themselves as learners rather than capable clinicians:

The convenor summarised the case and then asked residents in the audience for their ideas about a differential diagnosis. Junior residents answered in low‐volume voices and employed an interrogative upturn of pitch at the end of their sentences to indicate that their responses were questions rather than statements in response to the convenor's questions. (H2T1 Fieldnotes)

Consultants and supervisors did most of the talking in IM case conferences sharing perspectives in back‐and‐forth exchanges while residents listened.

The conversation jumped to and fro between the consultants who discussed the management of aspergillosis. They cited statistics and facts from sources of evidence and used experiential narratives to make their points. The junior doctors took a largely passive role as expert knowledge was talked into the space over their heads by consultants engaging in a game of evidential pinball. (H2T1 Fieldnotes)

Senior clinicians positioned themselves as knowledgeable and clear sighted users of evidence, employing language that appeared to marginalise the messiness and complexity of the medical practice that we had observed in both hospitals.

### 3.5 Informal identity work in teacher–learner interactions

Learners and supervisors used ‘set piece’ performances to figure their practices and author their identities. Surgical set pieces were performances of surgical craft in theatre whereas in IM, the typical ‘set piece’ performance was a case presentation.

#### 3.5.1 Learning in theatre

You wouldn't really be thinking right as a surgeon if you didn't jump at the opportunity to spend more time in theatre. It's much better than ward rounds and outpatients. Any registrar worth his salt would want to spend more time in here. (H1 T2 resident interview)

Figuring oneself as a surgeon and becoming accepted meant mastering surgical craft in theatre.

It's pressurised when you first meet a new consultant, because you could do something stupid, like you may not be able to cut a stitch because the scissors are blunt …. There's a big gulf between talking the talk and walking the walk, so there's usually a lot of anxiety going to a new job to demonstrate that you are already able to do something. (H1 T2 resident interview)

To practise their craft, trainees needed to present themselves as surgically capable under the gaze of the supervisor.

It's exactly like an old fashioned apprentice and master kind of thing. There are set pieces that you are expected to be able to do before you can move on. In the first instance they will watch you do something simple, like tie a knot, hold an instrument. That kind of stuff would tip them off as to whether you are technically proficient. As an assistant anticipating the next move, you know, focusing the camera correctly, showing them you are interested, focused on the operation, if you understand what the next step is. These are all hints. (H1 T2 Resident interview)

Surgical teachers typically used both verbal and physical means of communication to author themselves as coaches. Their talk indicated what trainees should do, see, and be careful about.

Can you see the vessels there? Now I want to keep that one because it's important, but that one we can zap. I think it's important because I think it looks after this bit of the gut that were going to keep, so we need to be careful of that. I don't care about bleeding from the mucosa, I care about bleeding from the mesentery. (H1 T2 Fieldnote)

In addition to providing verbal and physical guidance, surgeons authored a cultural imperative to keep moving on. This meant that surgical trainees had to practise their skills under considerable time pressure. ‘Come on, cut cut cut, let's get on with it’ (H1 T2 Field note).

Unlike internists, surgeons gave regular feedback. During surgical procedures, this feedback was often direct and unambiguous. ‘He is very hard on the criticism, but it is all constructive while you are operating’ (H2 T2 Resident interview). Surgical teachers also communicated implicit feedback by, for example, taking over control of a procedure from a resident.

I don't explicitly say anything when I'm taking over. But what a surgical trainee will understand by that, is that we've come to an area where I'm not happy for him to proceed directly himself. I'm not entirely happy with his understanding of the basis of what we are doing and therefore I'll take over. It's unsaid, but that's always the way it's been in surgical training. (H2T2 C interview)

Becoming a surgeon in theatre was about recognising and reproducing the practices of surgical supervisors, while authoring oneself as a surgeon and learning to talk knowledgeably in expected ways:

I have to admit I saw Mr X's' way of putting in the umbilical port, I did not particularly like it, but I do not actually know any other way. I've seen other people do different ways that look a lot simpler. But while I work with X I'll do it his way …. Everybody says to you ‘while you are working for me I want you to do it this way’. When you come to your own appointment, you pick up the bits that you liked from everybody, you do it your way. (H1 T2 Resident Interview)

#### 3.5.2 Case presentation in internal medicine

Case presentation provided a critical opportunity to self‐author as a capable physician in IM teams.

You just want to give the salient points to the consultant and see that he is happy to go along with the plan …. He doesn't need the full story. All he needs is a couple of bullet points and what we are doing. We're just selling him the story. …. If I'm presenting the story to the consultant, I think I'm looking to see does he agree with me. Has he any more to add? I'm not looking for validation or a pat on the back. (H2 T1 Resident Interview)

IM trainees figured themselves as physicians by comparing salient details that they chose to emphasise in their case presentation to those selected by their consultant supervisors:

If you admit someone and then you go through it with the consultant, and if they pick up on things that you missed, it's a really good learning experience, and if they picked out the same things, it's really satisfying that you think that you've done it properly. (T1 H1 Junior resident interview)

Senior team members listened for coherence and the inclusion of detail that allowed them to make sense of cases in junior doctors' case presentations:

I have listened to poor referrals. I think ‘Would you get to the point; what is the real issue here?’ You don't want to start off with ‘This man, he had a double by‐pass, he had a heart attack, and he is on Furosemide’. You want to say ‘He's got chest pain’ and then I'm thinking ‘Is he a high risk or a low risk patient?’ Your wheels are turning, and when you hear he has had a by‐pass you think ‘Well ok, how is his ecg?’ Whereas if you don't really know what the problem is and you are hearing the other information first, you can't, follow it. You need to see how they arrived at the impression. Once you can see how they arrived at their conclusion, it is usually fine. (T1 H2 resident interview)

Thus, case presentation in IM was about self‐authoring as capable of identifying salient detail, anticipating questions in listeners' minds and assembling a coherent abstract representation of the patient's presentation which made clear the rationale for conclusions or actions.

Rather than coach learners' thinking, internal medicine supervisors often sought to interactively align residents' case presentation to their own thinking and habits.

Resident *She's a 56‐year‐old lady*.

Consultant (Interrupts). *Is she a new patient?*

Resident *She is new‐ish. She was last seen in 2010*.

Consultant *She's a re‐referral then? Is it asthma then? Has she ever smoked?*

Resident *She has smoked*. *She had non‐Hodgkin's T‐Cell lymphoma in 2000 treated with CHOP and radiation*

Consultant (Interrupts*). To where, her chest?*

Resident *Chest yeah. Her CT in 2014 showed some upper lobe changes that they thought were associated with radiation therapy, but in her most recent chest x‐ray they do not appear to be any apical changes. She feels her symptoms started after she got radiation treatment*. H1 T1 Video transcript, resident case presentation in OPD

Teachers in internal medicine positioned themselves as models more than as coaches during informal interactions with learners, for example, by giving verbal demonstrations (cognitive broadcasts) of their thinking for their trainees. By articulating what they could see and were thinking, they modelled the practice of internal medicine:

You just think out loud about something; you show your way of reasoning … It's so much more interesting than just reading a book and following procedure. I think that hearing how someone is reasoning is very interesting. (H1 T1 Consultant interview)

Consultant monologues were the predominant form of teaching in internal medicine teams:

The consultant asks to see the patient's chest x‐ray. When the x‐ray appears on the screen, he talks about what he sees. He says that he thinks that there are bilateral pleural effusions on the chest x‐ray because there is loss of the normal diaphragmatic shadow and yet the left heart border is clearly visible. He then checks that his team understands the tasks that need to be carried out. (H1 T1 Field note)

## 4 DISCUSSION

Our principal finding was that the specialty‐specific practices of surgery and IM determined what was taught, how it was taught and what was learned. The roles of clinical teacher and learner were particular to each specialty and culturally anchored.^31^ Teachers and learners negotiated their identities within cultural worlds characterised by specialty‐specific ways of knowing, talking and being.

These observations align strongly with current descriptions of workplace learning where identity formation is characterised as a negotiation between learners' engagement with the affordances of the workplace, and its demands of them.^46^, ^47^, ^48^ Our work suggests that clinical teams should be conceptualised as cultural worlds in which agency is negotiated dialectically in relation to the socio‐historic norms and expectations of the team as well as the parent specialties.^49^ Learning in specialty‐specific cultural worlds not only limits learners' agency but promotes the reproduction of culturally sanctioned practices and dispositions.^13^, ^50^ Becoming a surgeon or an internist is therefore situated in a contested space between the demands of workplace contexts, specialty cultures and the aptitudes and aspirations of individual learners.^48^ Contextual threats to learner centredness and the perpetuation of dominant culturally scripted teaching practices and identities represent important challenges for faculty developers who wish to address the deficiencies of clinical education. There is good evidence, for example, that learners who can exercise agency to harness the available dialogue and practices in working or learning contexts can greatly enhance their learning experiences.^51^ Our work suggests that the solutions to the acknowledged problems of clinical education need to be founded on well informed constructions of the particular discourses that apply in specialty‐specific cultural worlds.

Becoming a specialist represents a form of identity work shaped by participation in clinical teams and the alignment of self with the historical values, practices and traditions of a particular discipline.^32^, ^34^ Identity work describes how individuals create, claim, discard and negotiate social and role identities in relation to others in social contexts.^52^ In this study, we found that learners in formal and informal clinical learning contexts used strategies of narrative positioning and self‐authoring to discursively make identity claims as surgeons and IM physicians. They did so in relation to discipline specific cultural backdrops of valued ways of knowing, talking and so forth that were embedded in the everyday working practices of internal medicine physicians and surgeons. Explorations of clinical education have highlighted the centrality of work in defining the curriculum of clinical education in PGME.^2^, ^53^ Given that it is work that defines what is learned and how it is learned, Morris and Swanwick^2^ suggest that clinical supervisors should select and structure meaningful work opportunities for trainees, articulate key features of the workplace hidden curriculum and provide working and learning environments that enable belonging.^2^, ^53^ This study and our previous work^19^ suggest that these clinical supervisor roles were for the most part not actualised in the service driven clinical workplaces we observed. These deficiencies could be addressed through faculty development, but only if faculty developers are prepared to acknowledge the cultural specificity of how clinical education is enacted in different specialties and the particularities of the cultural worlds where teaching, learning and working occur.

### 4.1 Recommendations for faculty development

Given the profoundly situated nature of the curriculum of the workplace, and the particularities of teaching and learning in different specialties, faculty developers could greatly enhance their effectiveness by shifting emphasis from attempts to ‘improve’ clinical education using solutions derived from educational orthodoxy, toward facilitating change founded on contextual understandings. To achieve this, faculty developers need to work with clinical teachers, (using action research approaches for example), to observe and make sense of the workplace settings and teaching practices that they hope to change. Faculty developers should collaborate with clinicians to develop contextual curriculums that are customised for the politics and realities of the places where clinicians work.^54^ Video reflexive ethnography, (VRE) has been shown to be a very effective approach in helping clinicians to appreciate cultural, taken for granted aspects of context and practice.^55^ Technologies such as VRE could be used to resource participatory action research designs whereby clinicians collaborate directly in building understanding of workplace contexts and therefore solutions to the educational problems that pertain there.

### 4.2 Limitations

Although these findings could help design faculty development programs that are better suited to the realpolitik of workplace education, there are some limitations to their applicability. Whereas this study was strengthened by gathering data from two clinical teams in each of two separate teaching hospitals, features such as team structure, hierarchy and training curriculums were particular to one country. To increase the transferability of the findings, we used Figured Worlds as a strong analytical framework and treated the sites where we conducted the research and the specialties of surgery and IM as instances of clinical workplace learning. Transferability was assured, further, by the composition of the research team. The authors come from three different countries, the Netherlands, Ireland and the United Kingdom; they are all experienced in international medical education; and the team includes an IM physician, a psychologist and a general practitioner, whose specialty training was very different from either surgery or IM. Nonetheless, given the importance of understanding the work, workplaces and the contextual curriculums that shape clinical education, we recommend further research in other countries and different specialties. In keeping with the traditions of social anthropology, a single observer undertook the field work in this study. To minimise bias he kept a reflexive diary and corroborated his observations using ‘on the hoof’ informal interviews with participants and formal exit interviews at the end of each observation period. The single observer effect was further mitigated by close cooperation, enriched by the different perspectives of the three researchers.

## 5 CONCLUSIONS

In clinical education, teacher and learner identity and the associated practices of teaching and learning are all shaped by, and contingent on, the sociocultural contexts in which clinical learning is situated. Participation in social structures such as specialty‐specific clinical teams, informs not only what is learned, but how it is learned. Clinical education is not, as it is sometime portrayed, a linear process of acquiring knowledge, but is instead a complex process of becoming, through mutual engagement in situationally specific curriculums of the workplace.^53^ Our work suggests that faculty development should move away from its current focus on expert led credentialing approaches to ‘fix’ clinical education and consider instead a more facilitative approach, founded on constructing shared understandings of workplace curriculums with the people who work there, the clinicians.

## CONFLICT OF INTERESTS

All authors have no competing interests in this research.

## ETHICS STATEMENT

Ethical approval was received from the Irish health services executive research ethics committees of both participating hospitals, that is, Galway University Hospital and Limerick University Hospital. The research ethics approval references were C.A. 1150 and 062/16 respectively. Preservation of anonymity and removal of any data that might identify participants, patients or people accidentally appearing in images were important issues in this ethnographic study. The research ethics committees in both cases were satisfied that the strategies employed to preserve anonymity were sufficient in this study.

## AUTHOR CONTRIBUTIONS

Peter Cantillon conceived and designed the study and carried out data collection, analysis and interpretation. He was primarily responsible for drafting the paper. He has approved the final version of this paper and is accountable for all aspects of the work. Willem de Grave participated in discussions of the conception and design of this study, reviewed raw data as these were collected, contributed to data analysis and interpretation and participated at all stages of the drafting process. He has given final approval for the version to be published and has agreed to be accountable for all aspects of the work. Tim Dornan contributed substantially to the conception and design of the work, reviewed raw data as these were collected, contributed to the data analysis and interpretation and was very actively involved at all stages of the drafting process. He approved the final version to be published this paper and has agreed to be accountable for all aspects of the work.
